# Delivery of high flow oxygen through nasal vs. tracheal cannulas: A bench study

**DOI:** 10.3389/fmed.2022.1068428

**Published:** 2023-01-12

**Authors:** Martin Cour, Claude Guérin, Florian Degivry, Laurent Argaud, Bruno Louis

**Affiliations:** ^1^Service de Médecine Intensive-Réanimation, Hospices Civils de Lyon, Hôpital Edouard Herriot, Lyon, France; ^2^Faculté de Médecine Lyon-Est, Université de Lyon, Université Claude Bernard Lyon 1, Lyon, France; ^3^Institut Mondor de Recherches Biomédicales, INSERM 955 CNRS 7000, Créteil, France

**Keywords:** work of breathing, weaning, high flow oxygen, nasal cannula, tracheal cannula, lung test

## Abstract

**Background:**

The use of high flow oxygen therapy (HFOT) has significantly escalated during the COVID-19 pandemic. HFOT can be delivered through both dedicated devices and ICU ventilators. HFOT can be administered to a patient *via* a nasal cannula (NC). In intubated patients, a tracheal cannula (TC) is used instead. In this study, we aim to compare the work of breathing (WOB) using a TC or NC and to explore whether differences exist among HFOT devices.

**Methods:**

Seven HFOT devices (three dedicated and four ICU ventilators) were connected to a manikin head (Laerdal Medical) through a NC (Optiflow 3S, large size, Fisher and Paykel Healthcare) or a TC (OPT 970 Optiflow+, Fisher and Paykel Healthcare). Each device was also attached to a manikin head that was connected to a lung simulator (ASL5000, Ingmar Medical), set at 40 ml/cmH_2_O compliance, 10 cmH_2_O/L/s resistance, and sinusoidal inspiratory effort (muscular pressure 10 cmH_2_O, rate 30 breaths/min). HFOT was delivered at 40 L/min and at 21% inspired oxygen fraction. The total WOB per breath and its resistive and elastic components were automatically analyzed breath by breath over the last 20 breaths by using Campbell's diagram.

**Results:**

The WOB and its resistive and elastic components were significantly lower with the TC than with the NC for every device, and systematically lower with the reference device than with others. These differences were, however, very small and may be not clinically relevant.

**Conclusion:**

The WOB is lower with the TC than with the NC and with the reference device, compared with the most recent devices.

## 1. Introduction

High flow oxygen therapy (HFOT) has increasingly been used in ICUs for acute hypoxemic respiratory failure in non-intubated patients (1). Its use has considerably increased during the COVID-19 pandemic (2). Methods to deliver HFOT have expanded, with several dedicated devices developed following on from historical devices (1) and HFOT implementation through ICU ventilators. Regardless of the device used, HFOT can be delivered through either nasal (NC) or tracheal (TC) cannulas. In a previous bench study, we found that the work of breathing (WOB) was marginally higher when devices (dedicated or ICU ventilators) were used, as compared to the reference historical Optiflow device (3).

The present study was motivated by comparison of the WOB between a NC or a TC, across various HFOT devices. A relevant clinical scenario to consider HFOT delivered through a TC or NC is upon tracheal cannula removal. In this case, it would be interesting to know whether a patient would be able to sustain a higher WOB through HFOT alone or would need additional respiratory muscle support compared with HFOT delivered through the TC. Another clinical scenario that the present study would like to address is the use of HFOT during spontaneous breathing trial in an intubated patient, and then after extubation. It has been shown that patients at low risk of extubation failure benefited from the use of NC-HFOT alone (4). In patients with a high risk of extubation failure, the effect of HFOT alone was uncertain (5). However, in those patients with a high risk of extubation failure, HFOT combined with non-invasive ventilation after extubation reduced the risk of reintubation, compared with HFOT alone (6). On the other hand, no data exist on the use of HFOT in the spontaneous breathing trial preceding extubation. We hypothesize that the WOB should be lower with a TC than with a NC because of the higher system flow-resistance of the latter. If verified, this finding would explain the excess in WOB after extubation under HFOT and, hence, would set out a rationale for supporting the respiratory muscles in addition to HFOT, in line with clinical evidence (6). We also explore whether differences exist between various HFOT devices.

## 2. Methods

**Figure 1:**
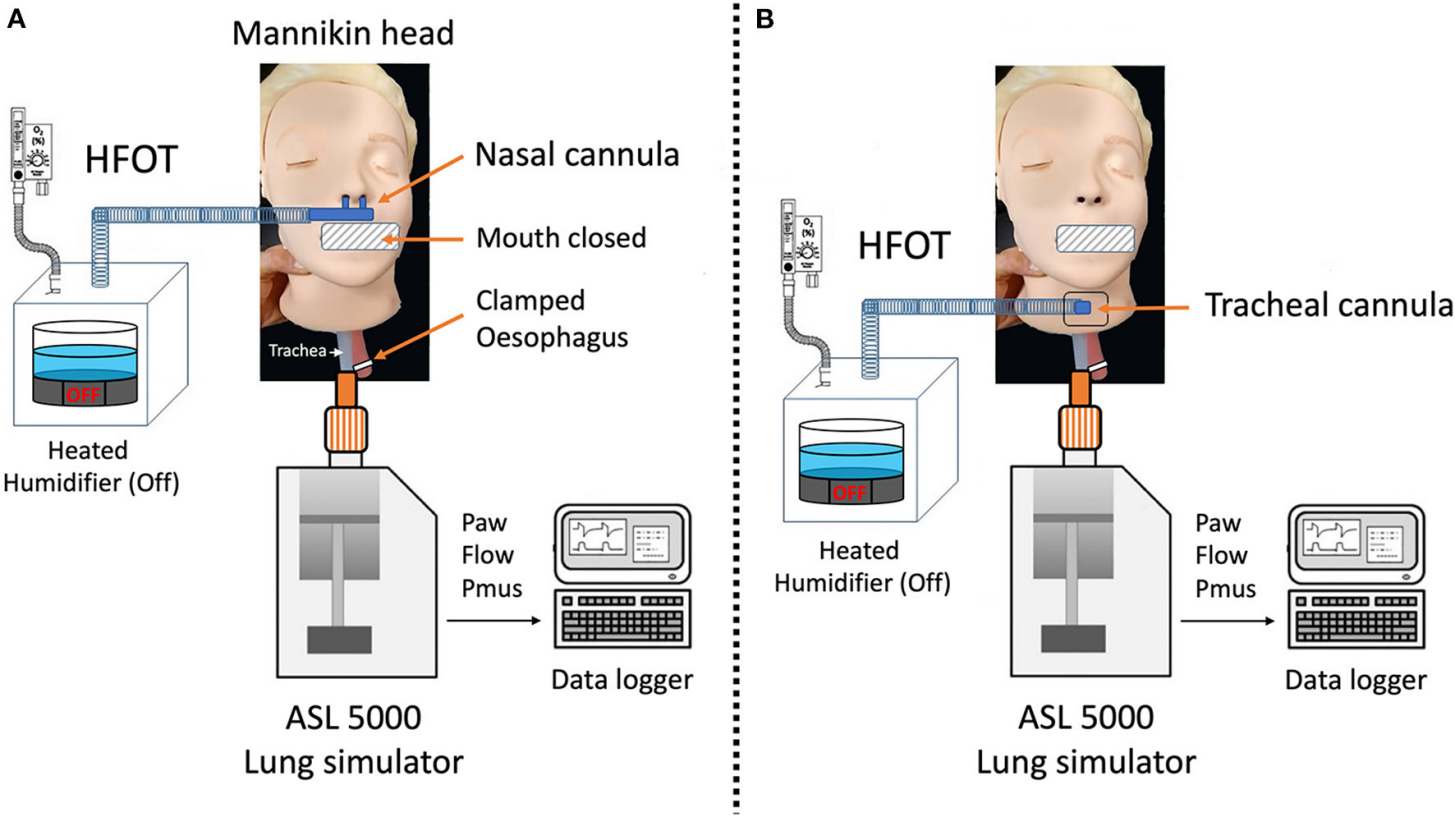
Bench model. High flow oxygen therapy (HFOT) devices were connected to the head of a manikin designed for cardiopulmonary resuscitation training through either a nasal cannula **(A)** or a tracheostomy cannula **(B)** directly inserted into a dedicated hole available in the neck of the manikin head. The mouth was kept closed and the esophagus was clamped. The trachea of the manikin was connected to a lung simulator (ASL5000, Ingmar Medical), set at 40 ml/cmH_2_O compliance, 10 cmH_2_O/L/s resistance, and sinusoidal inspiratory effort.

This study used previously reported data on HFOT with a NC (3). Seven HFOT devices (three dedicated: Optiflow Airvo2, Fisher and Paykel Healthcare, and HM80 BMC; and four ICU ventilators: T60, Air Liquide Medical Systems, V500, Draeger, V60 Plus, Philips, and G5, Hamilton Medical) were connected to a manikin head (Laerdal Medical) through a NC (Optiflow 3S, large size, Fisher and Paykel Healthcare) or a TC (OPT 970 Optiflow+, internal diameter 15 mm, Fisher and Paykel Healthcare) directly inserted into a dedicated hole in the neck of the manikin head (Figure 1). The mouth of the manikin head was kept closed to limit leaks for both the NC and TC (Figure 1). Each device was also attached to a manikin head which was connected to a lung simulator (ASL5000, Ingmar Medical), set at 40 ml/cmH_2_O compliance, 10 cmH_2_O/L/s resistance, and sinusoidal inspiratory effort (muscular pressure 10 cmH_2_O, rate 30 breaths/min) (Figure 1). HFOT was delivered at 40 L/min and at an inspired oxygen fraction of 21%, dry air. We selected a 40 L/min flow rate for HFOT because this value falls between the lower and upper limits of flows commonly used in clinical practice (i.e., 20 and 60 L/min) (7). The flow, airway pressure (Paw), and muscular pressure (Pmus) were measured by the ASL5000 and were acquired at 512 Hz for 2 min, with each device attached to either a NC or TC without and then with HFOT. The data were stored and analyzed off-line. The total WOB per breath and its resistive and elastic components were automatically analyzed breath by breath over the last 20 breaths by using the Campbell's diagram in Matlab (MATLAB 2019b, MathWorks), i.e., the volume–pressure curve, in which the volume was plotted on the y-axis and pressure on the x-axis. The plotted pressure was the difference between airway pressure and muscular pressure. We then fitted the lung compliance line to the volume–pressure data. The resistive and elastic components of the inspiratory total work of breathing in a single breath were measured as the area to the left and to the right of the compliance line, respectively. The esophageal pressure, i.e., muscular pressure, was input to the lung simulator. We chose a sinusoidal inspiratory effort with an amplitude of 10 cmH_2_O to simulate “medium” effort. The WOB per breath was then multiplied by the respiratory rate and expressed as J/min. On the same breaths, the inspired tidal volume (VTI) and positive end-expiratory pressure (PEEP) were also determined. The VTI was obtained by the time-integral of flow between periods of zero-flow. The flow-resistance of the TC was also measured by recording the airway pressure and flow upstream of the TC, through which flows of 0.5 and 1 L/s were actuated.

The data are presented as medians (1st−3rd quartiles) and were compared using the non-parametric two-factor ANOVA Scheirer–RayHare test. GLM with quasi-Poisson regression, non-parametric Man–Whitney, or Wilcoxon tests were used to compare each device using R software (version 4.2.0.), with the Optiflow device taken as a control.

## 3. Results

In the absence of HFOT from all devices, the total WOB was significantly higher with the NC than with TC: 4.3 (4.3–4.4) and 3.8 (3.7–3.8) J/min, respectively (*P* < 0.001). The same was true for its resistive [2.8 (2.7–2.8) vs. 2.4 (2.4–2.4) J/min (*P* < 0.001)] and elastic components [1.6 (1.5–1.6) vs. 1.4 (1.4–1.4) J/min (*P* < 0.001)]. When HFOT was administered, over all devices, the total WOB was significantly lower than when no HFOT was applied and was still higher with the NC than with TC: 3.9 (3.8–3.9) vs. 3.5 (3.5–3.5) J/min (*P* < 0.001). Similar results were found for its resistive [2.5 (2.4–2.5) vs. 2.4 (2.4–2.4) J/min, *P* < 0.001] and elastic components [1.4 (1.4–1.4) vs. 1.3 (1.3–1.3) J/min, *P* < 0.001]. For the NC and TC, over all devices, the VTI was 0.207 (0.207–0.207) and 0.195 (0.194–0.195) L, respectively, at baseline (*P* < 0.001) and 0.196 (0.194–0.196) and 0.187 (0.187–0.189) L, respectively, with HFOT employed (*P* < 0.001 for all results). The corresponding values of PEEP were 0.04 (0.03–0.06) and 0.05 (0.04–0.08) cmH_2_O at baseline (*P* < 0.001) and 0.6 (0.06–0.06) and 0.50 (0.43–0.58) cmH_2_O with HFOT employed (*P* < 0.001). The detailed results for each device are presented in Table 1. There was a significant interaction between the cannulas and devices in terms of total, resistive, elastic, and total WOB (Table 1). The WOB was significantly lower with the TC than with the NC for every device (Table 1). The same was true for its resistive and elastic components. The WOB was systematically lower for the Optiflow reference device than for the other devices, in terms of total WOB and its resistive and elastic components. The flow resistance of the TC was 3.5 cmH_2_O/L/s at 1 L/s flow. There was a significant interaction between devices and cannulas in terms of the VTI and PEEP (Table 2). The VTI was marginally higher for the NC than the TC, although statistically significant for the devices compared with the reference (Table 2). The same was true for the PEEP.

**Table 1:** Work of breathing between devices and cannulas at baseline and under high flow oxygen therapy.

**Device**	**Baseline**		**HFOT**	
	**Nasal cannula**	**Tracheal cannula**	**Nasal cannula**	**Tracheal cannula**
	Resistive WOB^*^, ^**^		Resistive WOB^*^, ^**^	
Optiflow	2.7 (2.6–2.8)	2.4 (2.4–2.4)	2.4 (2.4–2.4)	2.2 (2.2–2.2)
Airvo2	2.6 (2.6–2.7)^††^	2.4 (2.4–2.4)	2.5 (2.5–2.5)^††^	2.2 (2.2–2.2)
C5	2.8 (2.8–2.8)^††^	2.4 (2.4–2.4)^††^	2.5 (2.5–2.5)^††^	2.2 (2.2–2.2)
HM80	2.8 (2.8–2.8)^††^	2.4 (2.4–2.4)	2.4 (2.4–2.4)^††^	2.2 (2.1–2.3)
T60	2.8 (2.8–2.8)^††^	2.4 (2.3–2.4)	2.5 (2.5–2.5)^††^	2.2 (2.2–2.2)
V500	2.8 (2.8–2.8)^††^	2.4 (2.4–2.4)	2.5 (2.5–2.5)^††^	2.2 (2.2–2.2)
V60	2.8 (2.8–2.8)^††^	2.4 (2.4–2.4)	2.4 (2.4–2.4)^††^	2.2 (2.2–2.3)^††^
	Elastic WOB^*^, ^**^, ^†^		Elastic WOB^*^, ^**^, ^†^	
Optiflow	1.6 (1.6–1.7)	1.4 (1.4–1.4)	1.4 (1.3–1.4)	1.3 (1.2–1.3)
Airvo2	1.7 (1.6–1.8)^††^	1.4 (1.4–1.4)	1.4 (1.3–1.4)^††^	1.3 (1.2–1.3)^††^
C5	1.6 (1.6–1.6)^††^	1.4 (1.4–1.4)	1.4 (1.3–1.4)^††^	1.3 (1.2–1.3)
HM80	1.5 (1.5–1.6)^††^	1.4 (1.3–1.4)^††^	1.4 (1.3–1.4)	1.3 (1.2–1.3)^††^
T60	1.6 (1.5–1.6)^††^	1.4 (1.3–1.4)^††^	1.4 (1.3–1.4)^††^	1.3 (1.2–1.3)
V500	1.5 (1.5–1.6)^††^	1.4 (1.4–1.4)	1.4 (1.3–1.4)^††^	1.3 (1.2–1.3)
V60	1.6 (1.6–1.6)^††^	1.4 (1.4–1.4)	1.4 (1.3–1.4)	1.3 (1.2–1.3)^††^
	Total WOB^*^, ^**^, ^†^		Total WOB^*^, ^**^, ^†^	
Optiflow	4.3 (4.3–4.3)	3.8 (3.7–3.8)	3.7 (3.7–3.8)	3.5 (3.5–3.5)
Airvo2	4.3 (4.3–4.3)	3.8 (3.7–3.8)	3.9 (3.9–3.9)^††^	3.5 (3.5–3.5)^††^
C5	4.4 (4.3–4.4)^††^	3.8 (3.8–3.8)	3.9 (3.9–4.0)^††^	3.5 (3.5–3.5)^††^
HM80	4.3 (4.3–4.3)^††^	3.8 (3.7–3.8)	3.8 (3.8–3.8)^††^	3.6 (3.5–3.6)^††^
T60	4.3 (4.3–4.4)	3.7 (3.7–3.7)^††^	3.9 (3.9–3.9)^††^	3.5 (3.5–3.5)^††^
V500	4.3 (4.3–4.3)	3.8 (3.8–3.8)^††^	3.9 (3.9–3.9)^††^	3.5 (3.5–3.5)^††^
V60	4.4 (4.3–4.4)^††^	3.8 (3.8–3.8)^††^	3.8 (3.8–3.8)^††^	3.5 (3.5–3.6)^††^

Values are medians (1st−3rd quartiles) and the unit is J/min.

HFOT, high flow oxygen therapy; WOB, work of breathing.

* P < 0.05 for cannula,

** P < 0.05 for device,

† P < 0.05 for interaction between cannula and device,

†† P < 0.05 vs. Optiflow.

**Table 2:** Inspired tidal volume and positive end-expiratory pressure across devices and cannulas at baseline and under high flow oxygen therapy.

**Device**	**Baseline**		**HFOT**	
	**Nasal cannula**	**Tracheal cannula**	**Nasal cannula**	**Tracheal cannula**
	**Inspired tidal volume (L)** ^*^, ^**^, ^†^		**Inspired tidal volume (L)** ^*^, ^**^, ^†^	
Optiflow	0.207 (0.207–0.207)	0.194 (0.194–0.194)	0.192 (0.192–0.192)	0.187 (0.187–0.187)
Airvo2	0.207 (0.206–0.207)^††^	0.194 (0.194–0.194)^††^	0.196 (0.196–0.196)^††^	0.187 (0.187–0.188)^††^
C5	0.207 (0.207–0.207)^††^	0.195 (0.195–0.195)^††^	0.197 (0.197–0.197)^††^	0.187 (0.187–0.187)
HM80	0.207 (0.207–0.207)^††^	0.195 (0.195–0.195)^††^	0.194 (0.194–0.194)^††^	0.189 (0.189–0.189)^††^
T60	0.207 (0.207–0.207)^††^	0.193 (0.193–0.193)^††^	0.196 (0.196–0.196)^††^	0.185 (0.185–0.185)^††^
V500	0.207 (0.207–0.207)^††^	0.195 (0.195–0.195)^††^	0.196 (0.196–0.196)^††^	0.187 (0.187–0.187)^††^
V60	0.207 (0.207–0.207)^††^	0.195 (0.195–0.195)^††^	0.194 (0.194–0.194)^††^	0.189 (0.188–0.189)^††^
	**PEEP (cmH**_2_**O)** ^*^, ^**^, ^†^		**PEEP (cmH**_2_**O)** ^*^, ^**^, ^†^	
Optiflow	0.0 (0.0–0.0)	0.0 (0.0–0.0)	0.9 (0.9–0.9)	0.9 (0.9–0.9)
Airvo2	0.0 (0.0–0.0)^††^	0.0 (0.0–0.0)	0.6 (0.6–0.7)^††^	0.6 (0.6–0.6)^††^
C5	0.1 (0.0–0.1)	0.0 (0.0–0.1)^††^	0.6 (0.6–0.6)^††^	0.4 (0.4–0.4)^††^
HM80	0.0 (0.0–0.0)	0.1 (0.1–0.1)^††^	0.6 (0.6–0.6)^††^	0.5 (0.5–0.5)^††^
T60	0.1 (0.0–0.1)^††^	0.0 (0.0–0.0)^††^	0.6 (0.6–0.7)^††^	0.5 (0.5–0.5)^††^
V500	0.0 (0.0–0.0)^††^	0.1 (0.0–0.1)^††^	0.6 (0.6–0.6)^††^	0.4 (0.4–0.4)^††^
V60	0.1 (0.1–0.1)^††^	0.1 (0.1–0.1)^††^	0.8 (0.7–0.8)^††^	0.5 (0.5–0.5)^††^

Values are medians (1st−3rd quartiles).

HFOT, high flow oxygen therapy.

* P < 0.05 for cannula,

** P < 0.05 for device,

† P < 0.05 for interaction between cannula and device,

†† P < 0.05 vs. optiflow.

## 4. Discussion

In line with our hypothesis, we found that the WOB was lower with the TC than with the NC, and this was true for every device. Both components of the WOB were similarly affected by the change in the cannula. Three considerations should be taken for the effect of HFOT and cannulas on WOB. If VTI is constant, the WOB should be lowest with the least resistive device because the driving pressure should be lower. At similar effort, and hence similar driving pressure, the most resistive device should lower the flow and hence the WOB. Finally, Paw and flow were measured at the ASL inlet and the WOB measured downstream of the cannula at the alveoli, providing that the conducting airways of the manikin can be considered negligible. In our setup, because the muscular pressure is constant, the lower WOB with the TC is explained by slightly, but significantly, lower values of VTI and PEEP with the TC than with the NC. The fact that PEEP was lower with the TC than with the NC is consistent with more leaks in the former. Although nasal leaks are present with the NC, these are of lower magnitude with the TC and of a different nature. The flow-resistance of a TC, which bypasses the nasal airway, was lower than the nasal airway resistance of a large human population (8). The nasal airway resistance of humans can be much higher than that of the manikin head. In this condition, the leaks of the NC are substantial and may explain failure of nasal HFOT in acute hypoxemic respiratory failure. This point deserves further consideration in the clinical realm. The importance of leaks in the NC and TC increase when higher flows are delivered during HFOT. It is unclear why VTI was lower with the TC than with the NC during HFOT. One may speculate that the delivery of HFOT acts as a jet whose direction is not perfectly oriented toward ASL input, producing leaks that hinder inspiration from the ASL. With the NC, the jet becomes nil once the nose is passed.

In humans, the pressure reported during HFOT is higher than that reported in our study, even with the mouth open (9). The lower airway pressures we found at end-expiratory can be explained by the airway resistance and also by the resistance of the equipment. Let us consider the recent data presented by Vieira et al. (10), who also used the ASL5000 connected to devices delivering HFOT. The authors set the airway resistance to 10 cmH_2_O/L/s and the compliance to 60 cmH_2_O/L. For the same flow of 40 L/min, Vieira et al. found that the average airway end-expiratory pressure was 2.5 cmH_2_O. The resistance of our equipment, as mentioned in the methods, is 3.5 cmH_2_O/L/s. For a flow of 40 L/min, the pressure drop through the equipment is then 2.3 cmH2O. Subtracting this from the value of airway end-expiratory pressure stated by Vieira et al. gives a value very close to our present value. We set the compliance at 40 cmH_2_O/L; i.e., lower than that set by Vieira et al. This means that elastic recoil is greater in our study and, hence, may also contribute to the lower end-expiratory airway pressure.

The differences between the TC and NC are very small and may be not clinically significant. This is because, on the bench, the measurements are highly reproducible, which, coupled with the large number of breaths analyzed, increases the power of the comparisons. The same remark can be applied to the differences between the devices, which are significant but very small.

The limitations of the present study are that it is a bench study and extrapolation of the data presented here to patients must be performed with great caution. In particular, the muscular pressure was kept constant between the TC and NC to allow for comparisons; however, in clinical practice, this may change. In addition, on the bench, no feedback is available on patient effort and the corresponding respiratory support. Another limitation is that we did not insert an endotracheal tube (or tracheostomy tube) into the manikin's trachea. Thus, we did not directly simulate the clinical scenarios in which HFOT is applied *via* a NC after extubation, or HFOT administered *via* a TC connected to an endotracheal tube. However, delivering HFOT through an endotracheal tube requires the same TC we used, without the tube in place. The only thing that then differs is the endotracheal tube resistance, which would be constant and easily modeled. The final drawback is that we conducted the experiments with the TC and NC at a single HFOT of 40 L/min.

## 5. Conclusion

The WOB is lower with the TC than with the NC and with the reference device, compared with the most recent devices.

## Data availability statement

The original contributions presented in the study are included in the article/supplementary material, further inquiries can be directed to the corresponding author/s.

## Author contributions

CG, MC, and BL: conception, design, acquisition, analysis and interpretation of data, drafted the work, substantially revised the draft, approved the submitted version, and agreed both to be personally accountable for the author's own contributions and to ensure that questions related to the integrity of any part of the work, even ones in which the author was not personally involved, are appropriately investigated, resolved, and the resolution documented in the literature. FD: design, acquisition, substantially revised the draft, approved the submitted version, and agreed both to be personally accountable for the author's own contributions and to ensure that questions related to the integrity of any part of the work, even ones in which the author was not personally involved, are appropriately investigated, resolved, and the resolution documented in the literature. LA: design, interpretation of data, substantially revised the draft, approved the submitted version, and agreed both to be personally accountable for the author's own contributions and to ensure that questions related to the integrity of any part of the work, even ones in which the author was not personally involved, are appropriately investigated, resolved, and the resolution documented in the literature. All authors read and approved the final manuscript.
